# Replacement of Vitamin E by an Extract from an Olive Oil by-Product, Rich in Hydroxytyrosol, in Broiler Diets: Effects on Liver Traits, Oxidation, Lipid Profile, and Transcriptome

**DOI:** 10.3390/antiox12091751

**Published:** 2023-09-12

**Authors:** Javier Herrero-Encinas, Nereida L. Corrales, Fernando Sevillano, Robert Ringseis, Klaus Eder, David Menoyo

**Affiliations:** 1 Departamento de Producción Agraria, Universidad Politécnica de Madrid, ETS Ingeniería Agronómica, Alimentaria y de Biosistemas, 28040 Madrid, Spain; j.herreroen@upm.es (J.H.-E.); nereida.lunac@alumnos.upm.es (N.L.C.); f.sevillano@alumnos.upm.es (F.S.); 2 Institute of Animal Nutrition and Nutrition Physiology, Justus Liebig University Giessen, Heinrich-Buff-Ring 26-32, 35392 Giessen, Germany; robert.ringseis@ernaehrung.uni-giessen.de (R.R.); klaus.eder@ernaehrung.uni-giessen.de (K.E.); 3 Center for Sustainable Food Systems, Justus Liebig University Giessen, Senckenbergstr. 3, 35390 Giessen, Germany

**Keywords:** vitamin E, hydroxytyrosol, broiler chicken, liver, antioxidant, transcriptomic, lipid metabolism

## Abstract

The study examines the effect of replacing vitamin E (VE) with a liquid obtained from alpeorujo, an olive oil by-product rich in hydroxytyrosol (HT), as an antioxidant in broiler chicken feeds on the gene expression, lipid profile, and oxidation in the liver. There were five diets that differed only in the substitution of supplemental VE (0 to 40 mg/kg with differences of 10 mg/kg) by HT (30 to 0 mg/kg with differences of 7.5 mg/kg). A linear decrease (*p* < 0.05) in α-tocopherol concentration in the liver was observed with the replacement of VE by HT. There were no significant changes in triglyceride, cholesterol, or TBARS concentrations. The hepatic transcriptome showed 378 differentially expressed genes between broilers fed HT15 (20 mg/kg VE and 15 mg/kg HT) and HT0 (40 mg/kg VE) diets (*p* < 0.05 and fold change less or higher than 1.3). Significant changes in cell cycle, cell nucleus activity, neuroactivity, and necroptosis pathways and functions were observed. It is concluded that the olive oil by-product, rich in HT, could be used to spare VE as an antioxidant in broiler diets without affecting liver lipid and TBARS concentrations. The differential gene expression analysis showed a potential role of olive polyphenols in enhancing the chicken immune response.

## 1. Introduction

Vitamin E (VE) supplementation has been used in poultry nutrition owing to its well-established antioxidant and immunomodulatory effects [1,2]. This lipid-soluble vitamin (mainly α-tocopherol) is able to prevent oxidative tissue damage due to the cell protection against free radicals [3]. To meet nutritional requirements and to prevent oxidative stress, diverse studies have proposed different VE concentration levels in broiler chicken diets. According to NCR [4], a minimum of 10 IU per kg of feed is the required level of VE to prevent physiological insufficiencies in broiler chickens. However, these basal requirements can vary with the physiological state of the bird and the different compositions and amounts of the vitamin in most ingredients used in practical diets. Therefore, the VE recommendations vary from 8.95 IU/kg (plus 0.9 IU per gram of polyunsaturated fat in feed) [5] to 80 IU/kg according to production objectives [6,7]. The use of VE in poultry diets has nutritional and economic boundaries because VE is synthetically produced, and the cost-benefit of VE is curtailed. Thus, the search for alternatives to VE, including natural compounds, remains important for poultry [8].

In recent years, the use of bioactive compounds derived from olive oil by-products, including the polyphenols tyrosol, hydroxytyrosol (HT), and oleuropein, amongst others, has been progressively identified as a beneficial supplement in the nutrition of humans and food-producing animals [9,10]. Considering previous evidence in broiler chickens, olive bioactive extracts exerted antimicrobial and anticoccidial activity, anti-inflammatory properties, and antioxidant capacity [11,12,13,14]. Regarding antioxidant activity, the use of polyphenolic powder from olive mill wastewater in broiler chicken drinking water increased antioxidant capacity and reduced oxidative stress [15]. Furthermore, HT absorption and metabolism are characterized as a fast process of binding to lipoproteins acting as cardiovascular and antioxidant protectors and then distribution in several organs, including the liver, where HT is oxidized, methylated, and methylated–oxidized [16,17].

The antioxidant system is strongly related to lipid metabolism in poultry. In terms of lipid metabolism, the avian liver is the main organ with more importance and requirements compared to mammals and is very sensitive to stressors that can disrupt animal homeostasis [18]. The unique characteristic of lipid absorption through the hepatic portal system in birds makes the liver especially vulnerable and susceptible to ectopic lipid deposition and the consequent metabolic stress [19]. Additionally, hepatic tissue damage and the presence of disease, including fatty liver, are related to poor and compromised antioxidant capacity in poultry [20]. Therefore, it is important to boost the antioxidant defenses of the animal by adding VE or natural compounds with antioxidant capacity to the feed [8]. The mechanisms of action of natural antioxidant compounds, such as those found in by-products of olive oil, must be better understood if they are to replace VE as an antioxidant in chicken feed. In addition, natural antioxidants may be helpful to spare VE or maximize the activity of the vitamin in the diet, either directly or through a synergistic method [21]. Given that polyphenols have a wide variety of metabolic effects, we hypothesized that substituting VE for the HT-rich by-product of olive oil, alperujo, could have a positive influence on gene expression, lipid profiles, and oxidation in the liver. To this end, we performed transcriptomic, lipid profile, and oxidation analysis in the livers of broiler chickens fed diets that differed only in the substitution of supplemental VE (0 to 40 mg/kg with differences of 10 mg/kg) by HT (30 to 0 mg/kg with differences of 7.5 mg/kg).

## 2. Materials and Methods

### 2.1. Experimental Design and Diets

The feeding trial was approved by the Ethics Committee of the Universidad Politécnica de Madrid (Permission No. 2021-001). All the experimental procedures and animal care followed the established Spanish Guidelines for the Care and Use of Animals in Research [22]. The trial was conducted at the Universidad Politécnica de Madrid experimental facilities (Agricultural Production Department, Madrid, Spain). A total of 560 one-day-old male Cobb 500 chickens were randomly distributed among 35 floor pens (0.96 × 1.50 m) with 16 birds each and fed their respective experimental diets. The environmental conditions of the barn during the experiment were controlled automatically, and bird management was as recommended for broilers under commercial conditions [23]. The experimental product tested in the present study was OLIVOX^®^ (provided by Nutrición y Gestión S.L. Badajoz, Spain), which is obtained from olive waste (alperujo) in an industrial patented procedure (ES2436626B1) in the olive oil extraction process. The patented procedure consists of a mechanical separation of alperujo, resulting in its partition into two phases: liquid and solid. The liquid fraction (OLIVOX^®^) was used in the present study, and it is characterized by high moisture (53.2%) and ash (18.2%) contents and contains a high concentration of polyphenols (23.6 mg eq. gallic acid per g of product). Prior to its use as an ingredient in the feed, the liquid fraction was absorbed in a silica carrier (SIPERNAT, Evonik Corporation, Parsippany, NJ, USA) in a proportion of 60 and 40%, respectively. The levels of HT established in this research were based on the concentrations used by other researchers who observed positive effects on performance and oxidative stability of meat when including 9.5 mg/kg HT in broiler diets [24]. The final mixture (OLIVOX^®^ in a silica carrier) was included in the experimental feeds to ensure the desired amount of HT (0, 7.5, 15, 22.5, and 30 mg of HT per kg of feed).

The experiment was conducted as a completely randomized design with 5 treatments (HT0, HT7.5, HT15, HT22.5, and HT30) that consisted of 5 levels of HT (from 30 to 0 mg HT/kg), with a difference of 7.5 mg/kg between diets, used in substitution of VE (from 0 to 40 mg VE/kg), with a difference of 10 mg/kg between diets. Broiler chickens were fed experimental pelleted diets ad libitum across 2 feeding phases, consisting of a starter phase from 1 to 21 days of age and a grower phase from 22 to 39 days of age. The diets were based on corn and soybean meal and were fed in crumble and pellet form. The starter and finisher diets, which did not include any supplemental VE, contained 13.7 and 14.5 mg/kg of basal VE, respectively. The vitamin–mineral premix used in the experimental diets did not include any VE.

Representative samples of the diets were ground using a hammer mill (Model Z-I, Retsch, Stuttgart, Germany) fitted with a 0.75 mm screen and analyzed for moisture by oven drying (method 930.15), total ash with a muffle furnace (method 942.05), and nitrogen (N) using the Kjeldahl method (method 988.05) [25]. Crude protein content was calculated as N × 6.25. The gross energy of the experimental diets was determined using an adiabatic bomb calorimeter (model 1356, Parr Instrument Company, Moline, IL, USA). The VE concentrations were analyzed by HPCL following the method of Rey et al. [26]. The polyphenolic compounds were extracted from the OLIVOX^®^ and silica combination [27], and the total content was determined as indicated by the Folin–Ciocalteu method [28]. In addition, the HT and tyrosol contents were determined by HPLC [27]. The concentration of total polyphenols was 16.4 mg equivalents of gallic acid per g of product, and the HT and tyrosol contents were 9.10 and 2.55 mg per g of product, respectively, meaning around 71 percent of total polyphenols. The ingredient composition and calculated and determined analysis of the experimental diets are shown in Table 1 and Table 2.

### 2.2. Sample Collection

At the end of the experiment, one chick from each pen was chosen at random, weighed, and euthanized using CO_2_ asphyxiation. The liver was excised and weighed, and small aliquots were snap-frozen in liquid nitrogen and stored at −80 °C for further analysis, including measurements of the concentrations of TBARS, total lipids, cholesterol, triglycerides, and vitamin E (α-tocopherol), and for RNA isolation and hepatic transcript profiling.

### 2.3. Hepatic Concentrations of TBARS, Total Lipids, Triglycerides, Cholesterol, α-Tocopherol

Concentrations of thiobarbituric acid reactive substances (TBARS) in liver tissue homogenates were determined according to Wong et al. [29] and Kostner et al. [30]. Tissue aliquots of 50 mg were homogenized with 10 mmol/l Tris-HCl buffer using a TissueLyser (Qiagen, Hilden, Germany) at 20 Hz for 6 min under a N_2_ atmosphere. The supernatant was incubated with the TBA reagent and orthophosphoric acid at 100 °C for 1 h. Subsequently, TBARS were extracted with methanolic NaOH, and excitation and emission were determined at 532 and 560 nm, respectively, with a fluorescence spectrometer (Tecan Infinite M200 Plate Reader, Mainz, Germany). TBARS concentrations were calculated using a standard curve prepared with tetraethoxy propane.

Total lipids in the liver were measured following the method of Segura and López-Bote [31]. Lyophilized samples (80 mg) were homogenized in dichloromethane:methanol (8:2, *v*/*v*) using a mixer mill (MM400, Retsch Technology, Haan, Germany). The final biphasic system was separated by centrifugation at 10,000 rpm. The extraction was repeated three times. The solvent was evaporated under a nitrogen stream, and lipids were dried by vacuum desiccation. The total lipid content was determined gravimetrically.

The concentration of triglycerides and cholesterol was determined from approximately 70 mg of liver aliquot. Total lipids from the liver tissue were extracted with a mixture of n-hexane and isopropanol (3:2 *v*/*v*) according to Hara and Radin [32]. The lipid extracts were dried under a nitrogen atmosphere, and the lipids were dissolved with chloroform and Triton X-100 (1:1, *v*/*v*) as described by Eder and Kirchgessener [33]. The concentrations of triglycerides and cholesterol were determined with Triglycerides Standard FS and Cholesterol FS enzymatic reagent kits (DiaSis, Holzheim, Germany), respectively.

The concentration of α-tocopherol in the liver was determined using the method described by Olivares et al. [34]. Briefly, samples were homogenized in a dibasic sodium phosphate buffer (0.054 M), adjusted to pH 7.0 using HCl, and blended for 1 min in absolute ethanol and hexane (3:2, *v*/*v*). The retinol and tocopherol upper layer was evaporated, and ethanol was dissolved prior to the HPLC analyses by reverse phase (HP 1100, provided with a diode array detector; Hewlett Packard, Waldbronn, Germany). To perform the separation, a LiCrospher 100 RP-18 column (Agilent Technologies GmbH, Waldbronn, Germany) was used, the mobile phase was methanol:water (97:3, *v*/*v*) at a 2 mL/min flow rate, and the detector was fixed at 292 nm for the detection of α-tocopherol.

### 2.4. RNA Extraction

Total RNA from frozen liver aliquots (15 to 20 mg) was isolated using TRIzol reagent (Invitrogen, Karlsruhe, Germany) according to the manufacturer’s protocol. Then, it was analyzed for quantity and quality using an Infinite 200 M microplate reader equipped with a NanoQuant plate (both from Tecan, Mainz, Germany). The A260/A280 ratio and the average RNA concentration of total RNA samples were 1.86 ± 0.02 and 0.49 ± 0.03 µg/µL (*n* = 30), respectively.

### 2.5. Microarray Analysis and Bioinformatic Analysis

The microarray analysis was carried out with six randomly selected samples of liver total RNA per group (*n* = 30). After checking RNA quality (RNA integrity number values and A260:A280 ratios were 6.76 ± 0.54 (mean ± SD) and 1.77 ± 0.10, respectively), liver RNA samples were prepared for microarray hybridization following the Applied Biosystems^TM^ GeneChip^TM^ Whole Transcript PLUS Reagent Kit User Guide (Thermo Fisher Scientific, Waltham, MA, USA) at the Genomics Core Facility, “KFB—Center of Excellence for Fluorescent Bioanalytics” (Regensburg, Germany). Samples of total RNA were processed using an Affimetrix GeneChip Array (Chicken Gene 1.0 ST), which represents 18,214 genes, according to the Applied Biosystems^TM^ GeneChip^TM^ Whole Transcript (WT) PLUS Reagent KIT User Guide (Thermo Fisher Scientific, Waltham, MA, USA). The processed GeneChips were scanned, and the obtained cell intensity files, which provided an individual intensity value for each probe cell, were made from the image data employing the Command Console Software (https://www.thermofisher.com/de/de/home/life-science/microarray-analysis/microarray-analysis-instruments-software-services/microarray-analysis-software/affymetrix-genechip-command-console-software.html (accessed on 5 September 2023)) (Affimetrix, Santa Clara, CA, USA). Additionally, the Applied Biosystem^TM^ Transcriptome Analysis Console (TAC, v.4.0.2) software (Thermo Fisher Scientific) was used to analyze the compressed array image files (CEL files), calculate the summarized probe set signals with the Robust Multichip Analysis algorithm, perform the fold change (FC) comparisons, and calculate the significance *p*-values (ANOVA). The microarray data were deposited in MIAME-compliant format in the NCBI’s Gene Expression Omnibus public repository [35] (GEO accession No. GSE168390).

Differences in the hepatic transcriptomes between transcripts for groups HT7.5 vs. HT0, HT15 vs. HT0, HT22.5 vs. HT0, and HT30 vs. HT0 with a Student *t*-test *p*-value < 0.05 and a FC <−1.3 and FC > 1.3 were defined as upregulated and downregulated, respectively. Identical or similar criteria were used in several recent studies [36,37,38]. Because the adjusted *p*-values for all transcripts were > 0.05, the selection of differentially expressed transcripts could not be applied using the Benjamini and Hochberg false discovery rate adjustment method.

Gene set enrichment analysis (GSEA) was performed with the identified differentially expressed transcripts in order to identify enriched Gene Ontology (GO) terms within the GO categories biological process, molecular function, and cellular component and Kyoto Encyclopedia of Genes and Genomes (KEGG) pathways using the DAVID 6.8 bioinformatics resource [39,40]. GO terms and KEGG pathways were defined as enriched if the *p*-value was < 0.05. GSEA was performed separately for the up- and downregulated transcripts.

### 2.6. Validation of Microarray Data Using qPCR Analysis

Microarray data of nine differentially expressed transcripts were validated by qPCR. For qPCR analysis, total RNA from the HT0 and HT15 groups was used (*n* = 6). The synthesis of cDNA and the qPCR analysis were performed with a Rotor-Gene Q system (Qiagen, Hilden, Germany) as Keller et al. [41] described. Gene-specific primers were synthesized by Eurofins MWG Operon (Ebersberg, Germany). Normalization was carried out using multiple reference genes (glyceraldehyde-3-phosphate dehydrogenase (GAPDH), dehydrogenase complex flavoprotein subunit A (SDHA), beta actin (BACT), and hypoxanthine phosphoribosyltransferase 1 (HPRT1)) according to Vandesompele et al. [42]. The primer characteristics are listed in Supplementary Materials: Table S1.

### 2.7. Statistical Analysis

All statistical analysis was conducted with SAS [43] (release 9.2; SAS Institute, Cary, NC, USA), with experimental diets as a fixed effect and individual animals as the experimental unit. All parameters were tested with Levene’s test for homoscedasticity and the Shapiro–Wilk test for the normal distribution. Differences among experimental treatments were analyzed using one-way analysis of variance (one-way ANOVA), followed by a Tukey’s post hoc test when the data were normally distributed and the variances were homogeneous. Regression analysis was used to measure the linear or quadratic response to the replacement of VE by HT extract in the diet. For all tests, a *p*-value < 0.05 was considered statistically significant.

## 3. Results

### 3.1. Hepatic Traits and TBARS, Triglyceride, Cholesterol, and α-Tocopherol Concentrations

The results of the liver traits and the concentrations of TBARS, total lipids, triglycerides, cholesterol, and α-tocopherol are shown in Table 3. The relative liver weight and liver total lipids were not affected (*p* > 0.05) by dietary experimental treatments. Furthermore, no significant differences (*p* > 0.05) were observed in the concentrations of TBARS, cholesterol, and triglycerides in the liver. However, the concentration of α-tocopherol in the liver was significantly higher (*p* < 0.05) in the HT0 group compared to all HT-supplemented treatments. Moreover, the α-tocopherol concentration linearly (*p* < 0.05) decreased with the reduction in vitamin E inclusion in the diets.

### 3.2. Liver Transcriptome

According to the filter criteria applied (*p* < 0.05; fold change > 1.3 and <−1.3), a total of 33, 378, 76, and 117 transcripts were identified as differentially expressed in the liver among the comparisons HT7.5 vs. HT0, HT15 vs. HT0, HT22.5 vs. HT0, and HT30 vs. HT0, respectively. In this regard, HT15 vs. HT0 was the most relevant comparison, and therefore, the bioinformatics analysis results were focused on this assessment, although the rest of the comparisons were also analyzed (Supplementary Materials: Table S2).

**Figure 1:**
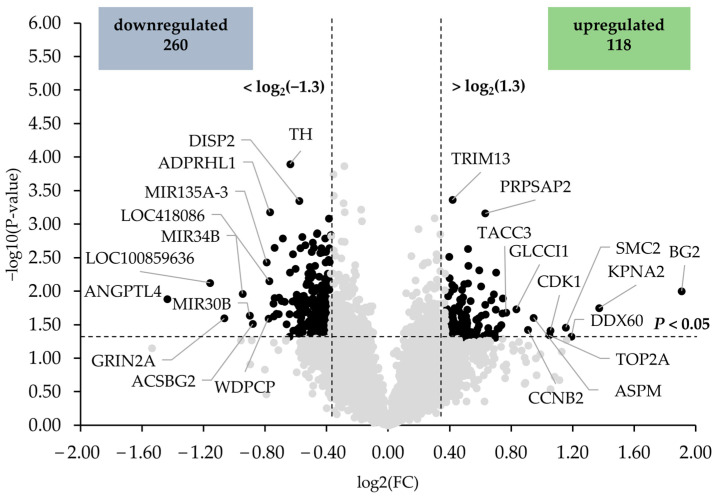
Volcano plot illustrating the differentially expressed transcripts in the liver of broiler chickens between HT15 and HT0 groups. The two filtering criteria are indicated by horizontal (*p*-value < 0.05) and vertical (fold change > 1.3 or <−1.3) dashed lines. Black dots represent the downregulated and upregulated transcripts.

In Figure 1, the differentially expressed transcripts between the HT15 and HT0 groups are illustrated as black dots in the volcano plot. Among the upregulated genes, only six (intestinal zipper protein (BG2), karyopherin alpha 2 (KPNA2), DEAD (Asp-Glu-Ala-Asp) box polypeptide 60 (DDX60), structural maintenance of chromosomes 2 (SMC2), cyclin-dependent kinase 1 (CDK1), and topoisomerase (DNA) II alpha 170 kDa (TOP2A)) exhibited a regulation >2.0-fold. The top 10 upregulated transcripts were, in decreasing order of their fold change (in brackets), as follows: BG2 (3.75), KPNA2 (2.58), DDX60 (2.28), SMC2 (2.23), CDK1 (2.08), TOP2A (2.07), asp (abnormal spindle) homolog (ASPM) (1.92), cyclin B2 (CCNB2) (1.88), glucocorticoid induced transcript 1 (GLCCI1) (1.78), and transforming, acidic coiled-coil containing protein 3 (TACC3) (1.70). Among the downregulated genes, three genes (angiopoietin-like 4 (ANGPTL4), RING finger protein 170-like (LOC100859636), and glutamate receptor, ionotropic, N-methyl D-aspartate 2A (GRIN2A)) were regulated <−2.0-fold. The top 10 downregulated transcripts were, in increasing order of their fold change, as follows: ANGPTL4 (−2.71), LOC100859636 (−2.23), GRIN2A (−2.09), microRNA 34b (MIR34B) (−1.93), microRNA 30b (MIR30B) (−1.87), acyl-CoA synthetase bubblegum family member 2 (ACSBG2) (−1.84), microRNA mir-135a-3 (MIR135A-3) (−1.73), WD repeat containing planar cell polarity effector (WDPCP) (−1.72), uncharacterized LOC418086 LOC418086 (−1.71), and ADP-ribosylhydrolase like 1 (ADPRHL1) (−1.70). The fold change and *p*-value of all differentially expressed transcripts between groups HT15 and HT0 are shown in Supplementary Materials: Table S3.

Microarray data of 10 differentially expressed transcripts between groups HT15 and HT0 were validated by qPCR. As shown in Table 4, the direction effect (positive or negative fold change) was the same among microarray and qPCR data for all validated transcripts, whereas the effect size (value of fold change) differed to some extent for the validated transcripts between qPCR and microarray data. Statistical analysis of qPCR data revealed that five genes were regulated either significantly (ANGPTL4, ACSBG2, hook microtubule tethering protein 1 (HOOK1), TOP2A, and SMC2) or at a *p*-value < 0.10 (GLCC1 and CDK1), whereas three genes (nuclear receptor subfamily 0 group B member 1 (NR0B1), TACC3, and CCNB1) were not significantly regulated.

**Figure 2:**
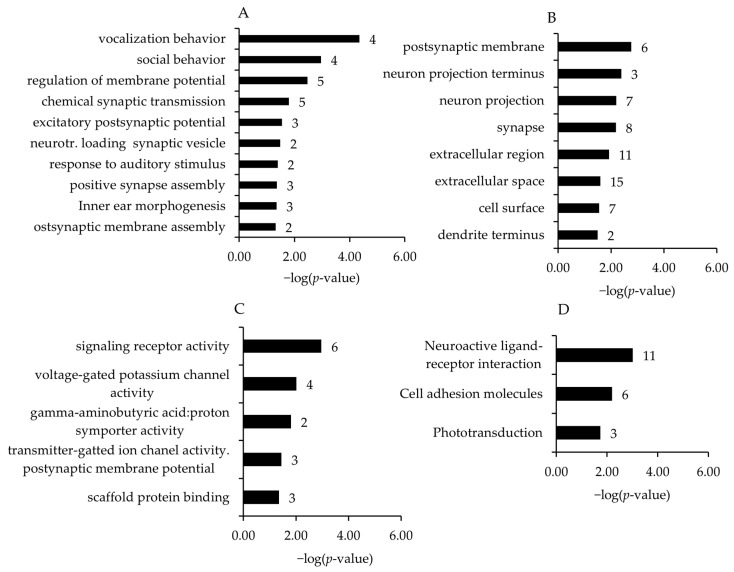
Most enriched gene ontology (GO) biological process (**A**), cell component (**B**), and molecular function (**C**) terms and Kyoto Encyclopedia of Genes and Genomes (KEGG) pathways (**D**) associated with the genes downregulated in broiler chickens between HT15 group and HT0 group. GO terms and KEGG pathways are sorted by the enrichment *p*-values (EASE score, *p*-value < 0.05) (top: lowest *p*-value; bottom: highest *p*-value). The number of genes is shown next to the bars.

To identify biological processes and pathways affected by the regulated transcripts, GSEA was performed using GO biological process, cell component, and molecular function terms and KEGG pathways. Due to the low number of regulated genes in the other comparisons, the present study was focused on the HT15 vs. HT0 comparison. Associated with the downregulated genes and within different GO and KEGG pathways, the four most enriched biological process terms assigned to the transcripts (in increasing order of their *p*-values) were vocalization behavior, social behavior, regulation of membrane potential, and chemical synaptic transmission (Figure 2A). For GO category cell component terms, the postsynaptic membrane, neuron projection terminus, neuron projection, and synapse were the most enriched terms (Figure 2B). For GO category molecular function terms, signaling receptor activity, voltage-gated potassium channel activity, gamma-aminobutyric acid:proton symporter activity, and transmitter-gated ion channel activity involved in the regulation of postsynaptic membrane potential were the most significant enriched terms (Figure 2C). The enhanced KEGG pathways assigned to the downregulated transcripts in the broiler chickens of the HT15 group were neuroactive ligand–receptor interaction, cell adhesion molecules, and phototransduction (Figure 2D).

**Figure 3:**
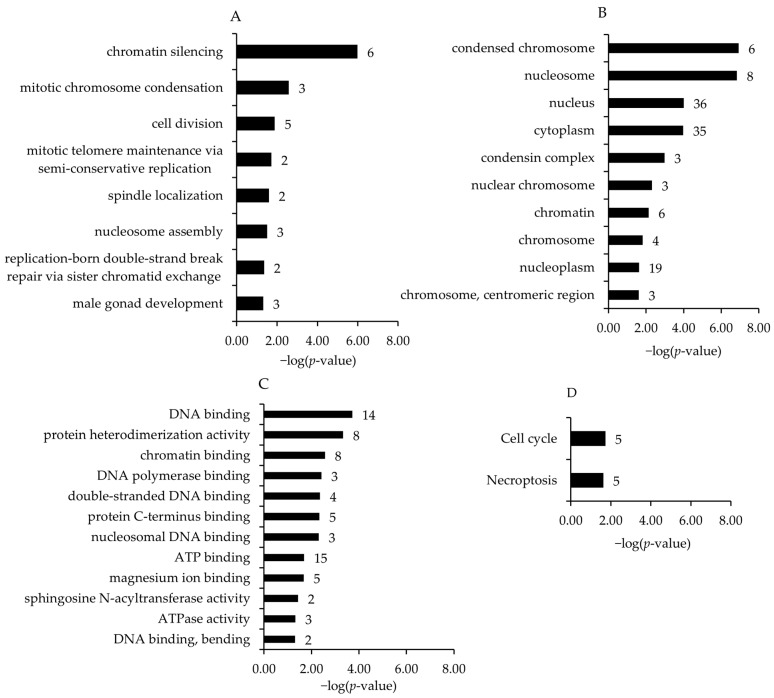
Most enriched gene ontology (GO) biological process (**A**), cell component (**B**), molecular function (**C**) terms and KEGG pathways (**D**) associated with the genes upregulated in broiler chickens between HT15 group and HT0 group. GO terms and KEGG pathways are sorted by the enrichment *p*-values (EASE score, *p*-value < 0.05) (top: lowest *p*-value; bottom: highest *p*-value). The number of genes is shown next to the bars.

Associated with the upregulated genes and within different GO and KEGG pathways, the four most enriched biological process terms assigned to the transcripts (in increasing order of their *p*-values) were chromatic silencing, mitotic chromosome condensation, cell division, and mitotic telomere maintenance via semi-conservative replication (Figure 3A). For GO category cell component terms, condensed chromosome, nucleosome, nucleus, and cytoplasm were the most enriched terms (Figure 3B). For GO category molecular function terms, DNA binding, protein heterodimerization activity, chromatin binding, and DNA polymerase binding were the most significant enhanced terms (Figure 3C). The most enriched KEGG pathways assigned to the transcripts downregulated in the broiler chickens of the HT15 group were cell cycle and necroptosis (Figure 3D).

## 4. Discussion

Plant polyphenols are naturally derived compounds with high antioxidant capacity. Righi et al. [44] recently reviewed studies with the aim of replacing dietary VE as an antioxidant with plant extracts in poultry. In most of the studies, extracts coming from grape, clove, green tea, or thyme showed positive effects on lipid metabolism by reducing plasma cholesterol compared to positive and negative VE controls [44]. Also, grape pomace, clove bud powder, and olive leaf extract reduced blood plasma MDA [44]. In addition, effects on liver size were not consistent among studies, with some of them not affecting and others decreasing the relative liver weight [44,45]. In the present study, supplementation with the olive-based extract rich in HT in replacement of VE had no significant effects on relative liver weight, total lipid, cholesterol, triglycerides, or TBARS concentrations. All the studies reviewed by Righi et al. [44] were performed with the aim of replacing VE supplemented in feeds at high doses, above 150 mg/kg. By contrast, our study was designed to replace basal VE as diets included from 40 to 0 mg/kg added VE. Therefore, our results suggest a similar effect of VE and HT on liver lipid concentrations when they are included as antioxidants at low doses in the feed. Moreover, the concentration of α-tocopherol in the liver was linearly reduced, which is in accordance with the VE reduction in diets. This was expected given the well-known direct correlation between dietary and liver α-tocopherol concentrations in broiler chickens [46]. Despite this VE reduction, the concentrations of TBARS were similar in the livers of broilers fed the experimental diets. Papadopoulou et al. [15] showed a reduction in TBARS in broiler chicken liver supplemented with polyphenols derived from olive mill wastewaters (OMW) containing 20 and 50 µg of polyphenols per ml of water. Likewise, pig and lamb studies observed that bioactive compounds derived from OMW reduced the levels of TBARS in plasma, liver, and other tissues, which implies that OMW can reduce lipid peroxidation [47,48]. Therefore, our data suggest a similar antioxidant effect of HT and VE on liver lipids.

The lack of effects on liver lipids and TBARS is consistent with the transcriptome results of the present study. The finding that only 33, 76, 378, and 117 annotated genes out of more than 18,000 screened were found to be differentially expressed (with filter criteria applied of fold change > 1.3 and <−1.3) between groups HT7.5 vs. HT0, HT15 vs. HT0, HT22.5 vs. HT0, and HT30 vs. HT0, respectively, suggests that VE substitution with HT extract had a slight influence on the hepatic transcriptome. The capacity of VE to regulate de novo fatty acid synthesis in the liver is widely known [49]. Studies like those of Huang et al. [50] and Mazur-Kuśnirek et al. [51] revealed that polyphenols (from green tea and rapeseed, respectively) have similar regulatory effects on lipid metabolism genes, which could explain the weak response observed in our study for the liver transcriptome. In a recent study, Korošec et al. [37] reported 594 differentially expressed genes, with a cut-off fold change of 1.2, when comparing the hepatic transcriptome of chickens fed 8.48 vs. 73.8 mg/kg of α-tocopherol. In our study, the comparison of HT0 and HT30 diets, which provided 11.3 and 48.9 mg/kg of α-tocopherol, respectively, retrieved only 117 differentially expressed genes with a similar cut-off fold change, suggesting again that the lower concentrations of VE in our study or the combination with HT reduces the effects on the transcriptome. However, it is noteworthy that the combinations of VE and HT resulted in different impacts on the liver transcriptome, with the HT15 diet promoting higher changes compared to the HT0 diet. The differentially expressed gene comparison between the HT15 and HT0 groups showed 118 upregulated and 260 downregulated transcripts, and thus, the rest of the discussion is focused on this comparison.

Bioinformatic GSEA of these 260 differentially downregulated hepatic genes revealed a particular involvement of the encoded proteins in biological process, cell component, and molecular function terms and KEGG pathways dealing with behavior, regulation of membrane potential, synapsis, and activity, as well as the connection and signaling of neurons. Interestingly, Xiao et al. [52] observed similar effects in broiler chickens (breast muscle) supplemented with an algae-based antioxidant compared to VE supplementation. In their study, transcriptome changes were related to nervous system development and function, connective tissue development, and auditory vestibular system development and function. Moreover, it is well known that HT is able to perform a neuroprotective role through protection against oxidative stress [17]. Thus, the bioinformatics results related to the neuronal modulation response shown in the present study might indicate that HT15 treatment induces a positive effect on the nervous system, but further studies are required to investigate this effect.

In addition, the bioinformatics GSEA of the 118 differentially upregulated transcripts showed that biological processes, molecular function terms, and KEGG pathways were related to chromatic silencing, condensed chromosome, cell cycle, DNA and ATP binding, or necroptosis. This agrees with the findings of Sabino et al. [53], who observed enriched GO terms related to condensed chromosome, condensed nuclear chromosome, and nuclear cell cycle activity on jejunum epithelial cells in broiler chickens supplemented with 300 mg/kg of an OMW in the feed. However, the study of Sabino et al. showed a downregulation of genes involved in lipid and cholesterol metabolism. Also, the study of Iannaccone et al. [54] showed a downregulation of cholesterol-metabolism-related genes in the blood of laying hens supplemented with 10% dried olive pomace. In the present study, the lack of a transcriptome effect related to lipid metabolism agrees with no significant changes in hepatic total lipids, cholesterol, or triglyceride concentration. Despite this, among the most differentially expressed genes, angiopoietin-like 4 (ANGPTL4), which is involved in the regulation of lipoprotein lipase activity and triglyceride metabolism [55], was −2.71-fold and −2.25-fold downregulated (transcriptomic and qPCR results, respectively) in HT15 vs. HT0. Also, the acyl-CoA synthetase bubblegum family member 2 (ACSBG2), which is strongly associated with abdominal fat deposition [56], was −1.84-fold (microarray) and −2.32-fold (qPCR) downregulated. The discrepancy between the clear effects in the above-mentioned studies related to lipid and cholesterol metabolism and the lack of response in our study might be related to the different diet and extract compositions or doses.

It is of interest that in the studies of Sabino et al. [53] and Iannaccone et al. [54], feeding olive-based extracts to healthy animals resulted in enriched GO term genes related to the regulation of viral genome replication and the inflammatory response. The most differentially upregulated transcripts in the present study were BG2, KPNA, and DDX60, 3.75-, 2.58-, and 2.28-fold, respectively. The gene BG2 is related to the chicken major histocompatibility complex, which is important in the immune response [57]. The gene KPNA2 (also known as importin alpha), besides its protein transport function, coordinates the stress response in the nucleus involving cellular apoptosis and necroptosis [58]. DDX60 is an antiviral helicase involved in interferon-inducible gene expression in response to viral infection [59]. Interferon-inducible gene expression is important in innate immunity against viruses as it triggers necroptosis to prevent virus replication [60]. Our results agree with those of Sabino et al. [53], who also reported an increased expression of antiviral genes, including KPNA2 and DDX60, in the jejunum of healthy broilers supplemented with OMW. As previously indicated, ANGPTL4 was downregulated in birds fed HT15 compared to HT0. Besides its role in lipid metabolism, ANGPTL4 has pleiotropic functions with anti- and pro-inflammatory properties [61]. The expression of ANGPTL4 is upregulated during the acute-phase response in mice challenged with LPS [62]. In addition, two microRNAs were significantly downregulated, microRNA 34b (−1.93-fold) and microRNA 30b (−1.87-fold). The downregulation of both microRNAs has been correlated to the host response to virus infection in broilers and goats through the modulation of genes involved in the interferon pathway and cytokine expression [63]. The upregulation of BG2, KPNA, DDX60, and necroptosis pathway and the downregulation of ANGPTL4, microRNA 34b, and microRNA 30b in the HT15-fed birds compared to H0 support the potential role of olive polyphenols in enhancing the innate immune response. Finally, it is interesting to notice that among the 260 downregulated genes, 38 were microRNAs, highlighting the epigenetic ability of olive polyphenols in the chicken liver and hence a way to explain their potential benefits through the control of gene expression [64].

## 5. Conclusions

Under the experimental conditions of this research, the substitution of up to 40 mg/kg of VE as an antioxidant in feeds with an olive oil by-product rich in HT had no effect on liver total lipid, triglyceride, cholesterol, or TBARS concentrations. The combination of 20 mg/kg of vitamin E and 15 mg/kg of HT affected the hepatic transcriptome by modulating the enriched GO terms related to cell cycle, activity in the cell nucleus, neuroactivity, and necroptosis. The differential gene expression analysis shows a potential role of olive polyphenols (mainly HT) in enhancing the chicken immune response.

## Figures and Tables

**Table 1:** Ingredient composition and calculated and determined analysis (% as feed basis) of the starter diets (0 to 21 d of age).

	HT0	HT7.5	HT15	HT22.5	HT30
**Ingredient**					
Corn	58.7	58.7	58.7	58.7	58.7
Soybean meal, 47% CP	36.4	36.4	36.4	36.4	36.4
Soy oil	1.20	1.20	1.20	1.20	1.20
Calcium carbonate	1.06	1.06	1.06	1.06	1.06
Monocalcium phosphate	1.02	1.02	1.02	1.02	1.02
DL-methionine, 99%	0.31	0.31	0.31	0.31	0.31
L-Lysine HCL, 78%	0.25	0.25	0.25	0.25	0.25
L-Threonine	0.09	0.09	0.09	0.09	0.09
L-Valine	0.04	0.04	0.04	0.04	0.04
Vitamin–mineral premix ^1^	0.55	0.55	0.55	0.55	0.55
Sodium chloride	0.38	0.38	0.38	0.38	0.38
OLIVOX^® 2^ mg/kg	0	824	1648	2473	3297
Vitamin E ^3^, mg/kg	40.0	30.0	20.0	10.0	0.0
**Calculated analysis**					
Moisture	12.3	12.3	12.3	12.3	12.3
AME_n_, kcal/kg	2920	2920	2920	2920	2920
Crude protein	21.9	21.9	21.9	21.9	21.9
Ether extract	3.93	3.93	3.93	3.93	3.93
Ash	5.50	5.50	5.50	5.50	5.50
Calcium	0.92	0.92	0.92	0.92	0.92
Digestible phosphorus	0.45	0.45	0.45	0.45	0.45
Sodium	0.16	0.16	0.16	0.16	0.16
Vitamin E, mg/kg	53.7	43.7	33.7	23.7	13.7
**Determined analysis**					
Moisture	12.2	12.5	11.7	11.6	11.8
Gross energy, kcal/kg	3938	3967	3981	3970	3962
Crude protein	22.5	22.7	22.6	22.7	22.5
Ash	5.07	4.70	5.09	4.92	5.08
Vitamin E ^4^, mg/kg	44.7	37.5	26.8	18.2	9.31

^1^ Supplied per kg of diet: vitamin A, 10,000 I.U; vitamin D3, 4800 I.U; vitamin K3, 3 mg; vitamin B1, 3 mg; vitamin B2, 9 mg; vitamin B6, 4.5 mg; vitamin B12, 40 μg; folic acid, 1.8 mg; niacin, 51 mg; pantothenic acid, 16.5 mg; biotin, 150 μg; Fe as iron (II) sulfate, 54 mg; Cu as copper (II) sulfate, 12 mg; Mn as manganous sulfate, 90 mg; I as potassium iodide, 1.2 mg; Zn as zinc oxide, 66 mg; Se as sodium selenite, 0.18 mg; 6-phytase EC 3.1.3.26, 10,000 FTU; calcium carbonate as a carrier. ^2^ OLIVOX^®^ (60%) in a silica carrier (40%). ^3^ DL-alpha-tocopheryl acetate added on top. ^4^ Alpha-tocopherol.

**Table 2:** Ingredient composition and calculated and determined analysis (% as feed basis) of the finisher diets (22 to 39 d of age).

	HT0	HT7.5	HT15	HT22.5	HT30
**Ingredients**					
Corn	64.0	64.0	64.0	64.0	64.0
Soybean meal, 47% CP	29.9	29.9	29.9	29.9	29.9
Soy oil	3.05	3.05	3.05	3.05	3.05
Calcium carbonate	0.96	0.96	0.96	0.96	0.96
Monocalcium phosphate	0.50	0.50	0.50	0.50	0.50
DL-methionine, 99%	0.28	0.28	0.28	0.28	0.28
L-Lysine HCL, 78%	0.23	0.23	0.23	0.23	0.23
L-Threonine	0.09	0.09	0.09	0.09	0.09
L-Valine	0.04	0.04	0.04	0.04	0.04
Vitamin–mineral premix ^1^	0.55	0.55	0.55	0.55	0.55
Sodium chloride	0.38	0.38	0.38	0.38	0.38
OLIVOX^® 2^ mg/kg	0	824	1648	2473	3297
Vitamin E ^3^, mg/kg	40.0	30.0	20.0	10.0	0.0
**Calculated analysis**					
Moisture	12.3	12.3	12.3	12.3	12.3
AME_n_, kcal/kg	3100	3100	3100	3100	3100
Crude protein	19.2	19.2	19.2	19.2	19.2
Ether extract	5.87	5.87	5.87	5.87	5.87
Ash	4.65	4.65	4.65	4.65	4.65
Calcium	0.77	0.77	0.77	0.77	0.77
Digestible phosphorus	0.34	0.34	0.34	0.34	0.34
Sodium	0.16	0.16	0.16	0.16	0.16
Vitamin E, mg/kg	54.5	44.5	34.5	24.5	14.5
**Determined analysis**					
Moisture	10.3	11.0	11.1	10.9	11.1
Gross energy, kcal/kg	4136	4103	4088	4117	4093
Crude protein	19.5	19.2	19.3	19.6	19.1
Ash	4.53	4.45	4.42	4.34	4.24
Vitamin E ^4^, mg/kg	48.9	35.7	26.0	19.0	11.3

^1^ Supplied per kg of diet: vitamin A, 10,000 I.U.; vitamin D3, 4800 I.U.; vitamin K3, 3 mg; vitamin B1, 3 mg; vitamin B2, 9 mg; vitamin B6, 4.5 mg; vitamin B12, 40 μg; folic acid, 1.8 mg; niacin, 51 mg; pantothenic acid, 16.5 mg; biotin, 150 μg; Fe as iron (II) sulfate, 54 mg; Cu as copper (II) sulfate, 12 mg; Mn as manganous sulfate, 90 mg; I as potassium iodide, 1.2 mg; Zn as zinc oxide, 66 mg; Se as sodium selenite, 0.18 mg; 6-phytase EC 3.1.3.26, 10,000 FTU; calcium carbonate as a carrier. ^2^ OLIVOX^®^ (60%) in a silica carrier (40%). ^3^ DL-alpha-tocopheryl acetate added on top. ^4^ Alpha-tocopherol.

**Table 3:** Effects of vitamin E ^1^ replacement by hydroxytyrosol in the diet on relative liver weight; total lipids; and concentrations of TBARS, cholesterol, triglycerides, and α-tocopherol in the liver of broiler chickens at 39 days of age ^2^.

Item ^3^	RLW, %BW	Total Lipids, %DW	TBARS, nmol/g	Chol, µmol/g	TG, µmol/g	α-Tocopherol, µg/g
HT0	2.07 ± 2.09	18.0 ± 0.1	69.6 ± 18.6	9.95 ± 0.97	20.3 ± 9.31	14.5 ^a^ ± 2.44
HT7.5	2.22 ± 2.41	17.3 ± 0.31	61.2 ± 13.1	9.57 ± 0.69	19.0 ± 2.40	10.4 ^b^ ± 2.11
HT15	2.24 ± 1.96	16.8 ± 0.26	68.6 ± 14.1	9.78 ± 1.32	13.4 ± 5.19	7.72 ^bc^ ± 1.72
HT22.5	2.19 ± 3.72	19.3 ± 0.18	69.9 ± 18.0	9.98 ± 0.70	22.2 ± 15.3	6.27 ^cd^ ± 2.02
HT30	2.15 ± 2.71	17.9 ± 0.25	73.5 ± 7.46	9.96 ± 1.52	23.1 ± 15.5	4.33 ^d^ ± 1.23
*p*-value ^4^						
Diet	0.71	0.37	0.70	0.96	0.58	<0.001
Linear	0.66	0.51	0.40	0.76	0.53	<0.001
Quadratic	0.20	0.73	0.43	0.68	0.27	0.055

^1^ DL-alpha-tocopheryl acetate. ^2^ Data are means ± standard deviation (*n* = 6 replicates for TBARS, Chol, and TG; *n* = 7 replicates for RLW, lipids and α-tocopherol). ^3^ RLW, relative liver weight; DW%, Dry Weight; TBARS, thiobarbituric acid reactive substances; Chol, cholesterol; TG, triglycerides. ^4^ Means with a different superscript (^a,b,c,d^) in the same row indicate a significant difference between groups (*p* < 0.05).

**Table 4:** qPCR validation of microarray data for selected differentially expressed transcripts between HT15 and HT0 groups ^1^.

	Fold Change		*p*-Value
	Microarray	qPCR	qPCR
ANGPTL4	−2.71	−2.25	0.020
ACSBG2	−1.84	−2.32	0.012
HOOK1	−1.67	−1.86	0.040
NR0B1	−1.68	−1.64	0.370
TACC3	1.70	1.18	0.126
GLCC1	1.78	1.17	0.082
CCNB1	1.88	1.25	0.216
TOP2A	2.07	1.26	0.050
CDK1	2.08	1.22	0.055
SMC2	2.23	1.32	0.026

^1^ Microarray and qPCR fold changes were calculated from *n* = 6 animals/group. ANGPTL4, Angiopoietin-like 4; ACSBG2, acyl-CoA synthetase bubblegum family member 2; HOOK1, hook microtubule tethering protein 1; NR0B1, nuclear receptor subfamily 0 group B member 1; TACC3, transforming acidic coiled-coil containing protein 3; GLCC1, glucocorticoid induced 1; CCNB1, cyclin B1; TOP2A, topoisomerase (DNA) II alpha; CDK1, cyclin dependent kinase 1; SMC2, structural maintenance of chromosomes 2.

## Data Availability

The data presented in this study are available on request from the corresponding author.

## Supplementary Materials

The following supporting information can be downloaded at: https://www.mdpi.com/article/10.3390/antiox12091751/s1. Table S1: Characteristics of gene-specific primers used for qPCR validation of microarray data. Table S2: Description of the up-regulated, down-regulated and total regulated transcripts between HT7.5 vs. HT0, HT15 vs. HT0, HT22.5 vs. HT0, HT30 vs. HT0 groups. Table S3: Fold change and p-value of all differentially expressed transcripts among HT15 and HT0 group.
