# Supplementary material for: Replacement of Vitamin E by an Extract from an Olive Oil by-Product, Rich in Hydroxytyrosol, in Broiler Diets: Effects on Liver Traits, Oxidation, Lipid Profile, and Transcriptome
Source: Antioxidants (Basel). 2023 Sep 12;12(9):1751. doi: 10.3390/antiox12091751 (PMC10525107; doi:10.3390/antiox12091751)
Supplement: Supplementary file 1 [file antioxidants-12-01751-s001.zip › antioxidants-2563570-supplementary.pdf]

Table S1. Characteristics of gene-specific primers used for qPCR validation of microarray data [1].

Gene	Forward (5` to 3`)<br>Reverse (5` to 3`)	Annealing temperature (ºC)	PCR product size (bp)	NCBI GenBank accession no.	Slope	R^2	E
Reference genes							
GAPDH	ACTGTCAAGGCTGAGAACGG<br>AGCTGAGGGAGCTGAGATGA	60	204	NM_204305.1	-3.49	0.997	1.93
SDHA	ATTCCCGTTTTGCCTACGGT<br>GGGAGTTTGCTCCAAGACGA	60	172	NM_001277398.1	-3.32	0.985	2.00
ACTB	ATGAAGCCCAGAGCAAAAGA<br>GGGGTGTTGAAGGTCTCAAA	60	223	NM_205518.1	-3.43	0.998	1.96
HPRT1	CAGCCCCTGCATCGTGATTG<br>GTGTCCTCCCATGCCCTTCA	60	169	NM_204848.1	-2.97	0.987	2.17
Target genes							
ANGPTL4	GCTTCAGCCACACCAACCAG<br>ATCACCGTCCAGCCACCTTC	60	190	XM_001232283.7	-3.22	0.995	2.05
ACSBG2	CGTTCGACGCTTGCAGATCC<br>CAGGACGTGAAGAGGACGCT	60	199	NM_001397597.1	-3.29	0.999	2.01
HOOK1	AGCTGAATCCAGCATCAGCAG<br>CTGGAGCGTTAGGCTCTTGTT	60	164	NM_001006542.2	-3.71	0.992	1.86
NR0B1	GGGCTTCCTGGCTAAGTGCT<br>GGCCAGGTAGATCCGGGTTG	60	95	NM_204593.2	-3.25	0.994	2.03
TACC3	CAGATCGTGTTGCGGGTCTG<br>AAAGCCACACTCTTCCGCTG	60	110	NM_001004429.4	-2.92	0.992	2.2
GLCC1	CCGGTGTACAGAGTGGACAGG<br>CGATGCTGAGCGCTGATGTG	60	225	NM_001012855.5	-3.44	0.995	1.95
CCNB1	GTTTGTGTAGAGCGGCGCAA<br>GCCAGGTCCTTTCGTAGCCT	60	249	NM_001004369.2	-3.55	0.986	1.91
TOP2A	ATGATGCTGCCATCACCCTG<br>GGCCTGGCTTCAAACCATCA	60	243	NM_204791.3	-3.99	0.974	1.78
CDK1	AGGCCAAGTGGTTGCAATGAA<br>GGTACAGCCTTGCATCCTGC	60	155	NM_205314.2	-3.43	0.998	1.96
SMC2	CCCTTAGTGGAGGTGCAAGC<br>TCTCCCACTGCTGCTTCAGA	60	177	NM_205230.2	-3.4	0.999	1.97

[1] GAPDH, glyceraldehyde-3-phosphate dehydrogenase; SDHA, dehydrogenase complex flavoprotein subunit A; ACTB, actin, beta; HPRT1, hypoxanthine phosphoribosyltransferase 1; ANGPTL4, Angiopoyetin-like 4; ACSBG2, acyl-CoA synthetase bubblegum family member 2; HOOK1, hook microtubule tethering protein 1; NR0B1, nuclear receptor subfamily 0 group B member 1; TACC3, transforming acidic coiled-coil containing protein 3; GLCC1, glucocorticoid induced 1; CCNB1, cyclin B1; TOP2A, opoisomerase (DNA) II alpha; CDK1, cyclin dependent kinase 1; SMC2, structural maintenance of chromosomes 2.

Table S2. Description of the up-regulated, down-regulated and total regulated transcripts between HT7.5 vs. HT0, HT15 vs. HT0, HT22.5 vs. HT0, HT30 vs. HT0 groups[1].

Comparison	Up-regulated transcripts	Down-regulated transcripts	Total regulated transcripts
HT7.5 vs. HT0	14	19	33
HT15 vs. HT0	118	260	389
HT22.5 vs. HT0	54	22	76
HT30 vs. HT0	57	60	117

Table S3. Fold change and *p*-value of all differentially expressed transcripts among HT15 and HT0 group [1].

Gene Symbol	Gene Description	FC	*p*-value
Downregulated			
ANGPTL4	angiopoietin-like 4	-2.71	0.013
LOC100859636	RING finger protein 170-like	-2.23	0.007
GRIN2A	glutamate receptor, ionotropic, N-methyl D-aspartate 2A	-2.09	0.025
MIR34B	microRNA 34b	-1.93	0.011
MIR30B	microRNA 30b	-1.87	0.023
ACSBG2	acyl-CoA synthetase bubblegum family member 2	-1.84	0.031
MIR135A-3	microRNA mir-135a-3	-1.73	0.004
WDPCP	WD repeat containing planar cell polarity effector	-1.72	0.025
LOC418086	uncharacterized LOC418086	-1.71	0.007
ADPRHL1	ADP-ribosylhydrolase like 1	-1.70	0.001
CCDC172	coiled-coil domain containing 172	-1.68	0.015
NR0B1	nuclear receptor subfamily 0, group B, member 1	-1.68	0.024
MIR1680	microRNA mir-1680	-1.67	0.002
HOOK1	hook microtubule-tethering protein 1	-1.67	0.013
MIR1779	microRNA mir-1779	-1.66	0.022
G0S2	G0/G1 switch 2	-1.64	0.022
MIR1655	microRNA mir-1655	-1.63	0.012
MIR135A2	microRNA 135a-2	-1.62	0.014
GUCA1C	guanylate cyclase activator 1C	-1.61	0.002
CYP2J6L3	cytochrome P450 2J6-like 3	-1.59	0.014
MIR551B	microRNA 551b	-1.58	0.031
MIR17	microRNA 17	-1.56	0.048
C5H14ORF38	chromosome 5 open reading frame, human C14orf38	-1.56	0.022
VIP	vasoactive intestinal peptide	-1.56	0.005
TH	tyrosine hydroxylase	-1.56	0.000
LOC771537	uncharacterized LOC771537	-1.54	0.014
CLEC3A	C-type lectin domain family 3, member A	-1.54	0.003
C5H14ORF38	chromosome 5 open reading frame, human C14orf38	-1.53	0.039
GRIA3	glutamate receptor, ionotropic, AMPA 3	-1.52	0.005
HS3ST5	heparan sulfate (glucosamine) 3-O-sulfotransferase 5	-1.51	0.013
ATOH7	atonal bHLH transcription factor 7	-1.51	0.011
PACRGL	PARK2 co-regulated-like	-1.51	0.022
LOC770479	gamma-aminobutyric acid receptor subunit beta-1-like	-1.51	0.037
MIR1718	microRNA mir-1718	-1.51	0.012
SLC32A1	solute carrier family 32 (GABA vesicular transporter), member 1	-1.51	0.015
PTPRQ	protein tyrosine phosphatase, receptor type, Q	-1.50	0.043
C8ORF34	chromosome 2 open reading frame, human C8orf34	-1.50	0.017
BRINP1	bone morphogenetic protein/retinoic acid inducible neural-specific 1	-1.49	0.015
DISP2	dispatched homolog 2 (Drosophila)	-1.49	0.000

Gene Symbol	Gene Description	FC	*p*-value
CALML3	calmodulin-like 3	-1.49	0.035
TCP10	t-complex 10	-1.49	0.014
FGF13	fibroblast growth factor 13	-1.49	0.020
BOLL	boule-like RNA-binding protein	-1.49	0.002
KCNH7	potassium voltage-gated channel, subfamily H (eag-related), member 7	-1.48	0.028
MIR128-2	microRNA 128-2	-1.48	0.024
MIR1722	microRNA mir-1722	-1.48	0.025
LOC769098	uncharacterized LOC769098	-1.48	0.012
NMS	neuromedin S	-1.48	0.030
CHRNA6	cholinergic receptor, nicotinic, alpha 6 (neuronal)	-1.47	0.002
ANO4	anoctamin 4	-1.47	0.012
MIR762	microRNA mir-762	-1.47	0.041
SOSTDC1	sclerostin domain containing 1	-1.46	0.012
CALR3	calreticulin 3	-1.46	0.012
TMEM240	transmembrane protein 240	-1.46	0.032
MIR1592	microRNA mir-1592	-1.46	0.021
NCAM2	neural cell adhesion molecule 2	-1.45	0.017
MIR1799	microRNA mir-1799	-1.45	0.017
MIR1598	microRNA mir-1598	-1.45	0.002
LOC101748624	uncharacterized LOC101748624	-1.45	0.013
MSLNL	mesothelin-like	-1.45	0.015
KCNQ3	potassium voltage-gated channel, KQT-like subfamily, member 3	-1.45	0.033
LOC420807	uncharacterized LOC420807	-1.44	0.008
LOC101749601	translation initiation factor IF-2-like	-1.44	0.008
MIR1741	microRNA mir-1741	-1.44	0.020
MIR301B	microRNA mir-301b	-1.43	0.015
FSTL5	follistatin-like 5	-1.43	0.005
ELOVL4	ELOVL fatty acid elongase 4	-1.43	0.014
ALG13	asparagine-linked glycosylation 13 homolog (S. cerevisiae)	-1.43	0.006
MIR106A	microRNA 106a	-1.43	0.028
MEPE	matrix extracellular phosphoglycoprotein	-1.43	0.028
FGF2	fibroblast growth factor 2 (basic)	-1.42	0.004
LOC100857478	uncharacterized LOC100857478	-1.42	0.047
NLGN1	neuroligin 1	-1.42	0.042
CNGA1	cyclic nucleotide gated channel alpha 1	-1.42	0.022
CHRM3	cholinergic receptor, muscarinic 3	-1.42	0.006
MIR1597	microRNA mir-1597	-1.42	0.008
SKOR2	SKI family transcriptional corepressor 2	-1.42	0.043
LOC428499	carboxymethylenebutenolidase homolog	-1.42	0.046
NLGN3	neuroligin 3	-1.42	0.036
LRRC9	leucine rich repeat containing 9	-1.41	0.023
CHRNA9	cholinergic receptor, nicotinic, alpha 9 (neuronal)	-1.41	0.003
PDCL2	phosducin-like 2	-1.41	0.022
ZP4	zona pellucida glycoprotein 4	-1.41	0.005
LOC422171	Rho GTPase activating protein 20-like	-1.41	0.010
FOXG1	forkhead box G1	-1.41	0.016
LOC423822	heparan sulfate glucosamine 3-O-sulfotransferase 1-like	-1.41	0.006
LOC101752022	maestro heat-like repeat-containing protein family member 2B-like	-1.41	0.013
KLHL31	kelch-like family member 31	-1.41	0.002
VGLL1	vestigial-like family member 1	-1.41	0.025
MIR202	microRNA 202	-1.41	0.014
LOC100857259	vesicular inhibitory amino acid transporter-like	-1.40	0.029
C1HXORF59	chromosome 1 open reading frame, human CXORF59	-1.40	0.040

Gene Symbol	Gene Description	FC	*p*-value
MIR9-1	microRNA mir-9-1	-1.40	0.045
LOC420300	lymphocyte antigen 6E-like	-1.40	0.006
BEGAIN	brain-enriched guanylate kinase-associated	-1.40	0.050
HCN1	hyperpolarization activated cyclic nucleotide-gated potassium channel 1	-1.40	0.025
GALNTL6	UDP-N-acetyl-alpha-D-galactosamine:polypeptide N-acetylgalactosaminyltransferase-like 6	-1.40	0.017
CXCL14	chemokine (C-X-C motif) ligand 14	-1.40	0.027
TMEM52B	transmembrane protein 52B	-1.40	0.002
STK32B	serine/threonine kinase 32B	-1.40	0.010
MIR19A	microRNA 19a	-1.40	0.043
GABRG3	gamma-aminobutyric acid (GABA) A receptor, gamma 3	-1.40	0.033
KIF25	kinesin family member 25	-1.39	0.010
LOC101749287	uncharacterized LOC101749287	-1.39	0.041
TPBG	trophoblast glycoprotein	-1.39	0.025
METAP1D	methionyl aminopeptidase type 1D (mitochondrial)	-1.39	0.016
PHEX	phosphate regulating endopeptidase homolog, X-linked	-1.39	0.017
SCRG1	stimulator of chondrogenesis 1	-1.39	0.017
WWP1	WW domain containing E3 ubiquitin protein ligase 1	-1.39	0.047
PRSS55	protease, serine, 55	-1.39	0.004
LOC101751393	PHD finger protein 7-like	-1.39	0.032
SERPINB10 CPOX	serpin peptidase inhibitor, clade B (ovalbumin), member 10 CPOX	-1.39	0.021
STEAP1	six transmembrane epithelial antigen of the prostate 1	-1.39	0.037
AADACL3	arylacetamide deacetylase-like 4-like	-1.39	0.050
AQP12	aquaporin 12	-1.38	0.026
OVST	ovostatin	-1.38	0.033
LTB4R	leukotriene B4 receptor	-1.38	0.038
LOC100859756	scale keratin-like	-1.38	0.023
MIR142	microRNA 142	-1.38	0.009
KBTBD12	kelch repeat and BTB (POZ) domain containing 12	-1.38	0.001
EGFL6	EGF-like-domain, multiple 6	-1.38	0.027
CLDN34	claudin 34	-1.38	0.038
PI15	peptidase inhibitor 15	-1.38	0.045
NTS	neurotensin	-1.38	0.014
ATP6V0D2	ATPase, H+ transporting, lysosomal 38kDa, V0 subunit d2	-1.38	0.030
CCDC92	coiled-coil domain containing 92	-1.38	0.029
LOC428538	epidermal retinol dehydrogenase 2-like	-1.38	0.001
LOC100857916	scavenger receptor cysteine-rich type 1 protein M130-like	-1.38	0.022
RGPD1	RANBP2-like and GRIP domain containing 1	-1.38	0.033
LOC770649	nucleophosmin-like	-1.37	0.027
PCTP	phosphatidylcholine transfer protein	-1.37	0.030
LRTM2	leucine-rich repeats and transmembrane domains 2	-1.37	0.014
GCM2	glial cells missing homolog 2 (Drosophila)	-1.37	0.029
DLX5	distal-less homeobox 5	-1.37	0.021
TAL2	T-cell acute lymphocytic leukemia 2	-1.37	0.016
FAM172BP	family with sequence similarity 172, member B, pseudogene	-1.37	0.017
MIR1462	microRNA mir-1462	-1.37	0.047
NEFH	neurofilament, heavy polypeptide	-1.37	0.036
LIN7A	lin-7 homolog A (C. elegans)	-1.37	0.050
OC3	osteocalcin-like protein OC3	-1.37	0.041
GREB1	growth regulation by estrogen in breast cancer 1	-1.37	0.019
EFHC2	EF-hand domain (C-terminal) containing 2	-1.37	0.017
MIR383	microRNA 383	-1.37	0.028
LOC771876	macrophage mannose receptor 1-like	-1.37	0.009

Gene Symbol	Gene Description	FC	*p*-value
NME8	NME/NM23 family member 8	-1.37	0.006
MIR21	microRNA 21	-1.36	0.020
CTD-2207O23.12	Uncharacterized protein [Source:UniProtKB/TrEMBL;Acc:M0QY47]	-1.36	0.026
MIR1570	microRNA mir-1570	-1.36	0.037
PCYOX1L	prenylcysteine oxidase 1 like	-1.36	0.022
MIR206	microRNA 206	-1.36	0.048
LOC101748060	uncharacterized LOC101748060	-1.36	0.032
LOC427400	uncharacterized LOC427400	-1.36	0.037
CHRDL2	chordin-like 2	-1.36	0.012
SPAG1	sperm associated antigen 1	-1.36	0.022
LOC424727	structure-specific endonuclease subunit SLX1-like	-1.36	0.003
CNTNAP2	contactin associated protein-like 2	-1.36	0.048
LRRC48	leucine rich repeat containing 48	-1.36	0.025
EPHA10	EPH receptor A10	-1.36	0.020
RSPH9	radial spoke head 9 homolog (Chlamydomonas)	-1.35	0.046
RSPH1	radial spoke head 1 homolog (Chlamydomonas)	-1.35	0.044
C4H4ORF45	chromosome 4 open reading frame, human C4orf45	-1.35	0.024
XG	Xg blood group	-1.35	0.019
LOC425001	uncharacterized LOC425001	-1.35	0.019
C24H11ORF1	chromosome 24 open reading frame, human C11orf1	-1.35	0.041
SH3RF3	SH3 domain containing ring finger 3	-1.35	0.002
CRISP2	cysteine-rich secretory protein 2	-1.35	0.033
KLHL29	kelch-like family member 29	-1.35	0.014
KIAA0319	KIAA0319	-1.35	0.043
BLVRA	biliverdin reductase A	-1.35	0.008
MIR130B	microRNA 130b	-1.35	0.024
LOC420156	perilipin 3-like	-1.34	0.003
MMRN1	multimerin 1	-1.34	0.026
MYO5C	myosin VC	-1.34	0.039
ZNF488	zinc finger protein 488	-1.34	0.014
TRIP11	thyroid hormone receptor interactor 11	-1.34	0.013
MOV10L1	Mov10l1, Moloney leukemia virus 10-like 1, homolog (mouse)	-1.34	0.039
RAMP1	receptor (G protein-coupled) activity modifying protein 1	-1.34	0.030
DMC1	DNA meiotic recombinase 1	-1.34	0.050
TDRD9	tudor domain containing 9	-1.34	0.039
LOC768596	uncharacterized LOC768596	-1.34	0.034
LOC101752049	uncharacterized LOC101752049	-1.34	0.017
MUC2	mucin 2, oligomeric mucus/gel-forming	-1.34	0.050
EPYC	epiphycan	-1.34	0.040
ULK4	unc-51-like kinase 4 (C. elegans)	-1.34	0.017
MMEL1	membrane metallo-endopeptidase-like 1	-1.34	0.048
C1ORF146	chromosome 8 open reading frame, human C1orf146	-1.34	0.010
MIR1670	microRNA mir-1670	-1.34	0.036
LOC101749470	uncharacterized LOC101749470	-1.33	0.018
KRT12	keratin 12 (Meesmann corneal dystrophy)	-1.33	0.009
MSLNL	mesothelin-like	-1.33	0.034
RIBC2	RIB43A domain with coiled-coils 2	-1.33	0.011
KCNA5	potassium voltage-gated channel, shaker-related subfamily, member 5	-1.33	0.046
MIR7-2	microRNA 7-2	-1.33	0.048
ASB14	ankyrin repeat and SOCS box containing 14	-1.33	0.011
MIR1-1	microRNA 1-1	-1.33	0.039
SRRL	serine racemase-like	-1.33	0.006
CCDC63	coiled-coil domain containing 63	-1.33	0.016

Gene Symbol	Gene Description	FC	*p*-value
GKN2	gastrokine 2	-1.33	0.002
LOC425462	malignant fibrous histiocytoma-amplified sequence 1 homolog	-1.33	0.017
C10ORF112	chromosome 2 open reading frame, human C10orf112	-1.33	0.004
RSPH4A	radial spoke head 4 homolog A (Chlamydomonas)	-1.33	0.003
KIAA1456	KIAA1456	-1.33	0.021
C6orf58	chromosome 6 open reading frame 58	-1.32	0.014
FAM19A1	family with sequence similarity 19 (chemokine (C-C motif)-like), member A1	-1.32	0.041
RIPPLY2	ripply transcriptional repressor 2	-1.32	0.040
SHISA6	shisa homolog 6 (Xenopus laevis)	-1.32	0.024
LOC421856	uncharacterized LOC421856	-1.32	0.032
LOC101751334	phosphoglucomutase-like protein 5-like	-1.32	0.048
FIBCD1	fibrinogen C domain containing 1	-1.32	0.046
LOC423277	galectin-related protein-like	-1.32	0.025
PIGQ	phosphatidylinositol glycan anchor biosynthesis, class Q	-1.32	0.043
MIR92A1	microRNA 92a-1	-1.32	0.008
PAQR9	progestin and adipoQ receptor family member IX	-1.32	0.013
PPP1R3B	protein phosphatase 1, regulatory subunit 3B	-1.32	0.029
CCDC83	coiled-coil domain containing 83	-1.31	0.046
ADGB	androglobin	-1.31	0.006
CRYBB1	crystallin, beta B1	-1.31	0.032
MIR490	microRNA 490	-1.31	0.046
KCNJ6	potassium inwardly-rectifying channel, subfamily J, member 6	-1.31	0.037
AVPR2	arginine vasopressin receptor 2	-1.31	0.005
LOC100858320	protein eyes shut homolog	-1.31	0.007
MIR429	microRNA 429	-1.31	0.028
MORN3	MORN repeat containing 3	-1.31	0.039
COMMD3	COMM domain containing 3	-1.31	0.003
NHEDC2	Na+/H+ exchanger domain containing 2	-1.31	0.021
NEK11	NIMA-related kinase 11	-1.31	0.035
STK17A	serine/threonine kinase 17a	-1.31	0.017
PLIN1	perilipin 1	-1.31	0.020
LOC100858427	feather keratin Cos1-1/Cos1-3/Cos2-1-like	-1.31	0.015
GGT7	gamma-glutamyltransferase 7	-1.31	0.021
KAZALD1	Kazal-type serine peptidase inhibitor domain 1	-1.31	0.014
EBF3	early B-cell factor 3	-1.31	0.037
CCDC148	coiled-coil domain containing 148	-1.31	0.006
LOC415312	uncharacterized LOC415312	-1.31	0.024
LOC101748152	uncharacterized LOC101748152	-1.31	0.041
CYP26B1	cytochrome P450, family 26, subfamily B, polypeptide 1	-1.31	0.038
NSUN7	NOP2/Sun domain family, member 7	-1.31	0.001
LOC100859545	potassium channel subfamily K member 9-like	-1.31	0.048
LOC427778	ectonucleoside triphosphate diphosphohydrolase 1	-1.31	0.043
VSTM4	V-set and transmembrane domain containing 4	-1.31	0.010
LOC101749925	uncharacterized LOC101749925	-1.30	0.030
LOC101750580	uncharacterized LOC101750580	-1.30	0.037
LOC101748060	uncharacterized LOC101748060	-1.30	0.002
CEP57L1	centrosomal protein 57kDa-like 1	-1.30	0.047
FBXO43	F-box protein 43	-1.30	0.041
DZANK1	double zinc ribbon and ankyrin repeat domains 1	-1.30	0.004
MIR1789	microRNA mir-1789	-1.30	0.040
TMEM150A	transmembrane protein 150A	-1.30	0.020
LOC100857840	disintegrin and metalloproteinase domain-containing protein 21-like	-1.30	0.017
LOC422353	synaptotagmin-like protein 2-like	-1.30	0.021

Gene Symbol	Gene Description	FC	*p*-value
MIR1658	microRNA mir-1658	-1.30	0.034
LOC427491	C2 calcium-dependent domain containing 4C-like	-1.30	0.048
B3GNTL2	UDP-GlcNAc:betaGal beta-1,3-N-acetylglucosaminyltransferase-like 2	-1.30	0.033
PIK3C2G	phosphatidylinositol-4-phosphate 3-kinase, catalytic subunit type 2 gamma	-1.30	0.025
SLC5A12	solute carrier family 5 (sodium/monocarboxylate cotransporter), member 12	-1.30	0.007
LOC421298	ankyrin repeat domain-containing protein 9-like	-1.30	0.021
LOC769834	trophoblast glycoprotein-like	-1.30	0.037
FYTTD1L	forty-two-three domain containing 1-like	-1.30	0.017
AGTR1	angiotensin II receptor, type 1	-1.30	0.001
KCNH7	potassium voltage-gated channel, subfamily H (eag-related), member 7	-1.30	0.024
Upregulated			
BG2	intestinal zipper protein	3.75	0.010
KPNA2	karyopherin alpha 2 (RAG cohort 1, importin alpha 1)	2.58	0.018
DDX60	DEAD (Asp-Glu-Ala-Asp) box polypeptide 60	2.28	0.047
SMC2	structural maintenance of chromosomes 2	2.23	0.035
CDK1	cyclin-dependent kinase 1	2.08	0.038
TOP2A	topoisomerase (DNA) II alpha 170kDa	2.07	0.045
ASPM	asp (abnormal spindle) homolog, microcephaly associated (Drosophila)	1.92	0.025
CCNB2	cyclin B2	1.88	0.038
GLCCI1	glucocorticoid induced transcript 1	1.78	0.018
TACC3	transforming, acidic coiled-coil containing protein 3	1.70	0.021
HIST1H46L2	histone cluster 1, H4-IV, germinal H4-like 2 (similar to human histone cluster 1, class H4 genes)	1.68	0.013
STMN1	stathmin 1	1.67	0.021
HIST1H2B7	histone cluster 1, H2B-VII (similar to human histone cluster 1, class H2B, member N)	1.66	0.032
HIST1H2B7L2	histone cluster 1, H2B-VII-like 2 (similar to human histone cluster 1, class H2B, member N)	1.66	0.032
MOV10	Mov10, Moloney leukemia virus 10, homolog (mouse)	1.64	0.036
CRHR2	corticotropin releasing hormone receptor 2	1.64	0.036
KIF14	kinesin family member 14	1.62	0.005
IRF8	interferon regulatory factor 8	1.62	0.019
HIST1H2BO	histone cluster 1, H2bo	1.62	0.049
DIAPH3	diaphanous-related formin 3	1.61	0.043
SPP1	secreted phosphoprotein 1	1.61	0.011
ICOS	inducible T-cell co-stimulator	1.59	0.014
NCAPG	non-SMC condensin I complex, subunit G	1.58	0.042
SMC4	structural maintenance of chromosomes 4	1.57	0.041
HIST1H2A4L2	histone cluster 1, H2A-IV-like 2 (similar to human histone cluster 2, class H2A, member C)	1.57	0.035
LOC101749238	histone H2A-IV-like	1.56	0.043
HIST1H2A4L2	histone cluster 1, H2A-IV-like 2 (similar to human histone cluster 2, class H2A, member C)	1.56	0.045
HIST1H2A3	histone cluster 1, H2A, III (similar to human histone cluster 2, class H2A, member C)	1.56	0.045
RAD51	RAD51 recombinase	1.55	0.022
HIST1H2A4	histone cluster 1, H2A, IV (similar to human histone cluster 2, class H2A, member C)	1.55	0.048
PRPSAP2	phosphoribosyl pyrophosphate synthetase-associated protein 2	1.55	0.001
TSPAN8	tetraspanin 8	1.55	0.016
HIST1H2A4L3	histone cluster 1, H2A-IV-like 3 (similar to human histone cluster 2, class H2A, member C)	1.54	0.046
KIF23	kinesin family member 23	1.52	0.023
C11ORF34	chromosome 24 open reading frame, human C11orf34	1.52	0.008
SNORD12C	small nucleolar RNA, C/D box 12C	1.51	0.005
SLC41A2	solute carrier family 41, member 2	1.50	0.032

Gene Symbol	Gene Description	FC	*p*-value
SIDT1	SID1 transmembrane family, member 1	1.50	0.025
DOCK9	dedicator of cytokinesis 9	1.50	0.049
PCNA	proliferating cell nuclear antigen	1.49	0.037
MIR1596	microRNA mir-1596	1.49	0.040
ACTR3	ARP3 actin-related protein 3 homolog (yeast)	1.49	0.026
TYMS	thymidylate synthetase	1.47	0.050
LOC100858439	keratin, type I cytoskeletal 14-like	1.46	0.041
USH1C	Usher syndrome 1C (autosomal recessive, severe)	1.45	0.014
HELLS	helicase, lymphoid-specific	1.44	0.044
LGALS8	lectin, galactoside-binding, soluble, 8	1.43	0.004
SYNGR3	synaptogyrin 3	1.43	0.039
CTD-2370N5.3	Uncharacterized protein [Source:UniProtKB/TrEMBL;Acc:J3QR06]	1.43	0.002
RFK	riboflavin kinase	1.43	0.008
TMEM41B	transmembrane protein 41B	1.43	0.019
CERS6	ceramide synthase 6	1.43	0.030
SNORD20	small nucleolar RNA, C/D box 20	1.43	0.033
SDC1	syndecan 1	1.43	0.030
ADH6	alcohol dehydrogenase 6 (class V)	1.42	0.027
BUB3	BUB3 mitotic checkpoint protein	1.42	0.034
MIR199B	microRNA mir-199b	1.42	0.024
HMGB2	high mobility group box 2	1.42	0.046
THYN1	thymocyte nuclear protein 1	1.42	0.027
DEK	DEK proto-oncogene	1.41	0.008
CERS1	ceramide synthase 1	1.40	0.049
HIST1H111L	histone cluster 1, H1.11L (similar to human histone cluster 1, class H1 genes)	1.40	0.038
ACE2	angiotensin I converting enzyme 2	1.40	0.033
HN1	hematological and neurological expressed 1	1.40	0.012
BFAR	bifunctional apoptosis regulator	1.39	0.010
LDHA	lactate dehydrogenase A	1.39	0.015
DUSP5	dual specificity phosphatase 5	1.39	0.023
CAPSL	calcyphosine-like	1.39	0.022
LGALS2	lectin, galactoside-binding, soluble, 2	1.39	0.024
H2AFZ	H2A histone family, member Z	1.39	0.030
PTMA	prothymosin, alpha	1.39	0.047
KCNMA1	potassium large conductance calcium-activated channel, subfamily M, alpha member 1	1.38	0.013
DSTN	destrin (actin depolymerizing factor)	1.38	0.042
PSEN1	presenilin 1	1.38	0.033
HMGN2	high mobility group nucleosomal binding domain 2	1.38	0.016
SWAP70	SWAP switching B-cell complex 70kDa subunit	1.38	0.020
SOCS1	suppressor of cytokine signaling 1	1.37	0.032
HMGN5	high mobility group nucleosome binding domain 5	1.37	0.046
RRM1	ribonucleotide reductase M1	1.36	0.049
RAD21	RAD21 cohesin complex component	1.36	0.036
TRAF3	TNF receptor-associated factor 3	1.35	0.032
CDC20	cell division cycle 20	1.35	0.009
MRPS18C	mitochondrial ribosomal protein S18C	1.35	0.031
ACP1	acid phosphatase 1, soluble	1.35	0.018
CD82	CD82 molecule	1.35	0.029
PTMS	parathymosin	1.34	0.031
LOC101751423	uncharacterized LOC101751423	1.34	0.009
SLC25A29	solute carrier family 25 (mitochondrial carnitine/acylcarnitine carrier), member 29	1.34	0.034
DSG2	desmoglein 2	1.34	0.026

Gene Symbol	Gene Description	FC	*p*-value
KITLG	KIT ligand	1.34	0.020
UVRAG	UV radiation resistance associated	1.34	0.049
CXADR	coxsackie virus and adenovirus receptor	1.34	0.023
TRIM13	tripartite motif containing 13	1.34	0.000
MTPN	myotrophin	1.33	0.008
ANXA5	annexin A5	1.33	0.035
MTFR2	mitochondrial fission regulator 2	1.33	0.036
IDH3A	isocitrate dehydrogenase 3 (NAD+) alpha	1.33	0.029
DNA2	DNA replication helicase/nuclease 2	1.33	0.029
ABCB1LB	ATP-binding cassette, sub-family B (MDR/TAP), member 1-like B	1.33	0.041
COMT	catechol-O-methyltransferase	1.33	0.032
C4H4ORF29	chromosome 4 open reading frame, human C4orf29	1.33	0.026
PKHD1	polycystic kidney and hepatic disease 1 (autosomal recessive)	1.33	0.032
DYNLRB2	dynein, light chain, roadblock-type 2	1.32	0.010
HPGDS	hematopoietic prostaglandin D synthase	1.32	0.006
MLXIP	MLX interacting protein	1.32	0.033
DCAF5	DDB1 and CUL4 associated factor 5	1.32	0.035
PRKAR2A	protein kinase, cAMP-dependent, regulatory, type II, alpha	1.32	0.003
RRAGB	Ras-related GTP binding B	1.31	0.032
TMEM229B	transmembrane protein 229B	1.31	0.026
FANCD2	Fanconi anemia, complementation group D2	1.31	0.030
STARD3NL	STARD3 N-terminal like	1.31	0.020
LBR	lamin B receptor	1.31	0.012
CAP1	CAP, adenylate cyclase-associated protein 1 (yeast)	1.31	0.026
DENND1B	DENN/MADD domain containing 1B	1.30	0.018
ALDH1A1	aldehyde dehydrogenase 1 family, member A1	1.30	0.024
OAZ1	ornithine decarboxylase antizyme 1	1.30	0.023
TMBIM4	transmembrane BAX inhibitor motif containing 4 [Source:EntrezGene;Acc:51643]	1.30	0.033
CRYBG3	beta-gamma crystallin domain containing 3	1.30	0.037

[1] Microarray fold changes were calculated form n = 6 animals/group.
